# Efficacy of Pseudo‐Ceramide Absorption Into the Stratum Corneum and Effects on Transepidermal Water Loss and the Ceramide Profile: A Randomized Controlled Trial

**DOI:** 10.1111/jocd.16655

**Published:** 2024-11-04

**Authors:** Tomoki Akahane, Daisuke Watanabe, Eri Shimizu, Kosei Tanaka, Kazuhiro Kaizu

**Affiliations:** ^1^ Skin Care Products Research Laboratory Kao Corporation Odawara Kanagawa Japan; ^2^ Skin Care Products Research Laboratory Kao (China) Research and Development Center Co. Ltd Shanghai People's Republic of China; ^3^ Analytical Science Research Laboratory Kao Corporation Tochigi Japan

**Keywords:** ceramide profile, ceramides, pseudo‐ceramide, sensitive skin, skin, skin barrier

## Abstract

**Background:**

Changes in the level or profile of ceramides are associated with decreased stratum corneum (SC) barrier function. Topical application of a pseudo‐ceramide (pCer)‐containing moisturizer can improve barrier function. Additionally, pCer that absorbs into the SC may improve ceramide profiles.

**Aim:**

We investigated the relationship between pCer absorption into the SC and SC properties and determined the efficacy of a pCer‐containing spray compared with that of a commercial spray without pCer.

**Patients/Methods:**

Patients with self‐perceived dry and sensitive skin and decreased barrier function (transepidermal water loss [TEWL] > 10 g/m^2^h) were randomized into two groups to topically apply a pCer‐containing spray (test group; *N* = 33) or commercial spray without pCer (control group; *N* = 19) twice daily as a single‐blind study. SC function and ceramide properties were investigated before and after 4 weeks of application.

**Results:**

In the test group, the ceramide (NP)/(NS) ratio proportionally increased with the pCer application level after 4 weeks of pCer‐containing spray application. In the control group, there were no changes in SC function after topical application of the commercially available spray without pCer; however, the SC water content, TEWL, SC cell area, and scaling score improved in the test group. Furthermore, the changes in TEWL in the test group were significantly negatively correlated with the pCer application level.

**Conclusions:**

The efficacy of pCer‐containing sprays for those who have sensitive skin with impaired barrier function was demonstrated. Furthermore, the improvement in SC barrier function induced by pCer may contribute to normalizing the SC ceramide profile.

## 1 Introduction

Sensitive skin is characterized by uncomfortable sensations (e.g., pain and itchiness) in response to a normally insignificant stimulus [1]. Although the pathophysiological mechanism underlying sensitive skin remains unclear, it is hypothesized to be primarily associated with decreased barrier function and nervous hypersensitivity [2, 3]. Disrupted epidermal barrier, frequently observed under dry skin conditions such as atopic dermatitis and surfactant‐induced rough skin, is associated with the ceramide properties of the stratum corneum (SC) [4, 5]. Transepidermal water loss (TEWL)—an indicator of SC barrier function—is negatively correlated with endogenous ceramide levels in the SC, owing to the barrier function of the lipid bilayer that primarily consists of ceramides in the intercellular lipids of the SC [6, 7]. Furthermore, skin with decreased barrier function exhibits both quantitative and qualitative changes in ceramides [4, 7, 8]. Yokose et al. [9] reported that the ratio of ceramide (NP)/ceramide (NS)—ceramide subclasses—can be a marker for cutaneous barrier function because of a negative correlation between TEWL and the ceramide (NP)/(NS) ratio. A recent study revealed that people with skin highly sensitive to lactic acid irritation have a significantly lower ceramide (NP)/(NS) ratio than those without sensitive skin [10]. Therefore, while the endogenous ceramide level is decreased in the SC of atopic dermatitis in particular, the ceramide profile is deteriorated in the SC of both atopic dermatitis and sensitive skin. Ceramide supplementation is effective for skin with decreased barrier function and improves SC barrier and moisture‐retention functions [5, 11]. Ceramides can be supplemented as natural ceramides [5], pseudo‐ceramides [12], topically applicable ceramide precursors [13], or oral intake supplements [14]. In particular, pseudo‐ceramide (pCer)‐containing moisturizers exert an effect similar to that of natural ceramide‐containing moisturizers [5]; therefore, pCer, which is less expensive than natural ceramides, is being actively developed and evaluated.

**FIGURE 1:**
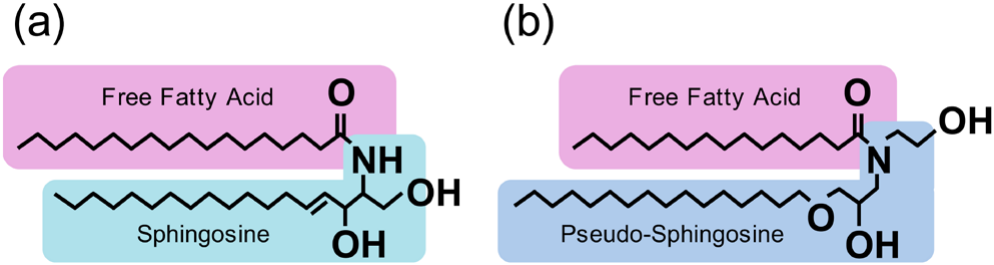
Chemical structure of natural ceramide and synthetic pCer. (a) Natural ceramide: Cer (NS). (b) Product name of pCer: SOFCARE CERAMIDE SL‐E (Kao Corporation), INCI Name: Cetyl‐PG hydroxyethyl palmitamide, Molecular. Weight: 598 g/mol (C_37_H_75_O_4_N), Melting point: 69.0°C–77.0°C, Purity > 97%. Cer (NS), ceramides containing non‐hydroxy fatty acids and sphingosines; pCer, pseudo‐ceramide.

Imokawa et al. developed cetyl‐PG hydroxyethyl palmitamide, a type of pCer that mimics the ceramide (NS) structure (Figure 1), and used it in moisturizers [4, 15, 16]. Safety studies of this ingredient were previously conducted [17, 18, 19]. Considering that pCer is optimized in structure and has the same moisturizing effect as natural ceramides, this raw material is called a ceramide functional ingredient [15]. The efficacy of moisturizers containing pCer has been demonstrated in skin with impaired barrier function, such as surfactant‐treated rough skin, atopic dermatitis skin, and sensitive skin with lactic acid irritation [5, 15, 16, 20, 21]. Nojiri et al. reported a substantial improvement in the SC function of participants with high sensitivity to lactic acid irritation when using a cream containing pCer and eucalyptus extract, which exerts a ceramide‐producing effect [20, 21]. These findings suggest the potential of formulations containing pCer to improve skin sensitivity. Additionally, in a study involving the continuous treatment of pCer‐containing lotion for atopic dermatitis with disrupted barrier function, the pCer that absorbed into the SC functioned in a manner similar to that of endogenous ceramides, resulting in a shift of the SC ceramide profile from atopic dermatitis to a healthy type [16]. Therefore, although insights into the contribution of pCer applied to the SC to improve the SC in atopic dermatitis skin have been provided, the relationship between the absorption of pCer into the SC and SC function as well as the endogenous ceramide properties in sensitive skin with impaired barrier function is yet to be demonstrated.

Recently, technologies for incorporating pCer into various skincare formulations have been developed, including the incorporation of pCer into a spray formulation that can be easily used throughout the body [22]. To incorporate a pCer into a low‐viscosity aqueous formulation, pCer should be purified, and crystallization should be suppressed using a small quantity of surfactant. Takagi et al. used a micromixer that achieves high‐speed mixing of pCer within milliseconds and achieved the industrial production of microdispersions of amorphous pCer [23]. This formulation exhibited higher pCer‐absorption ability than emulsified formulations prepared using general‐purpose pCer‐dispersion techniques [22]. Furthermore, Kawashima et al. [24] reported that this pCer‐containing spray can decrease itchiness and improve the water content in the SC of patients with atopic dermatitis.

In this study, we aimed to elucidate the influence of pCer absorption into the SC of participants with self‐perceived sensitive skin and impaired barrier function whose ceramide levels were not decreased but whose ceramide profiles were deteriorated on SC function and endogenous ceramide properties, with topical application of a pCer‐containing spray. Furthermore, to prove the efficacy of pCer‐containing spray for individuals with sensitive skin and impaired barrier function, we compared it with a commercially available spray formulation without pCer as a control product.

## 2 Materials and Methods

### 2.1 Study Design

In this study, healthy Japanese females (aged 20–49 years) had resided in Japan for at least 5 years and had judged themselves to have sensitive and dry skin conditions based on a questionnaire survey in which they were enrolled. Those who suffered from allergies such as rhinitis and atopic symptoms were eliminated by visual assessment conducted by the evaluators. To screen the eligible participants for this study, the TEWL of the cheek of 80 females was measured. Participants with skin diseases, including eczema and dermatitis, frequent drug users, pregnant females, and lactating females, were excluded. To evaluate the relation between pCer absorption into the SC and barrier function, 54 participants with TEWL > 10 g/m^2^h were included. Although subjects with high TEWL were gathered for this study, those with visible atopic dermatitis symptoms were excluded by the evaluators during selection, such that the inclusion of potential atopic dermatitis patients was unlikely. This study was conducted between February 28, and March 27, 2019. The participants were divided into two groups via randomization, considering that the differences in the mean TEWL and mean age between the groups were comparable (Table 1). Only the participants were blinded to the treatment allocation.

**TABLE 1:** Participant information.

	Test group	Control group
Number of participants	34	20
Age (years)^a^	35.4 ± 8.3	35.2 ± 9.6
Transepidermal water loss (g/m^2^h)^a^	19.7 ± 5.7	19.7 ± 3.8
Scaling score (visual assessment)	Normal: *n* = 16 (50.0%); Slight: *n* = 6 (18.8%); Moderate: *n* = 5 (15.6%); Marked: *n* = 3 (9.4%); and Severe: *n* = 2 (6.3%)	Normal: *n* = 10 (52.6%); Slight: *n* = 5 (26.3%); Moderate: *n* = 2 (10.5%); Marked: *n* = 2 (10.5%); and Severe: *n* = 0 (0%)

^a^ Mean ± SD.

As a washout procedure, both participant groups were asked to apply a washout lotion and washout emulsion on their entire face for 1 week to eliminate the influence of daily skincare; thereafter, the continuous use test phase was initiated. During the test phase, each participant applied a corresponding spray instead of the washout lotion twice daily for 4 weeks. A washout emulsion was continuously applied during the test phase. During the study period, the participants were not allowed to use any skin medication, change their daily cleansers, or add new products. At the beginning and after 4 weeks of the test phase, evaluations were performed via skin capacitance, TEWL measurements, collecting tape‐stripped SC, visual evaluation, and a questionnaire. One author conducted a blinded evaluation.

### 2.2 Materials

Table 2 presents a summary of the components of the lotion and emulsion used for the washout procedure. Table 3 presents a summary of the components of the test and control sprays.

**TABLE 2:** Components of the washout samples.

Washout lotion
Water/aqua, glycerin, butylene glycol, betaine, methyl gluceth‐20, polyethylene glycol‐32, polyethylene glycol‐60 hydrogenated castor oil, allantoin, succinic acid, arginine, methylparaben
Washout emulsion
Water/aqua, butylene glycol, glycerin, neopentyl glycol dicaprate, dimethicone, squalane, polysorbate 60, sorbitan stearate, sodium laureth‐4 phosphate, sodium methyl stearoyl taurate, carbomer, potassium hydroxide, cetyl alcohol, stearyl alcohol, methylparaben

**TABLE 3:** Components of the test and control spray.

Test spray
Water/aqua, glycerin, dipropylene glycol, cetyl‐PG hydroxyethyl palmitamide, butylene glycol, allantoin, cholesterol, sodium methyl stearyl taurate, polyethylene glycol‐60 hydrogenated castor oil, adipic acid, disodium ethylenediaminetetraacetic acid, arginine, Eucalyptus globulus leaf extract, phenoxyethanol
Control spray
Water/aqua, Nitrogen^a^

^a^ The control spray was a commercially available product.

### 2.3 Ceramide Assessment Method

SC samples were collected by stripping the right side of the cheek with 25 × 30‐mm square pieces of film‐masking tape 465 #40 (Teraoka Seisakusho, Tokyo, Japan). For each participant, three consecutive tape strips were used and each strip was divided into two parts. One part was used to analyze the total soluble protein level, and the other half was used to quantify ceramide and pCer. The intercellular lipids in the SC were extracted from one half of each tape sample using 1.8 mL of methanol/2‐propanol/chloroform mixture (9:9:2, v/v/v). Intracellular lipids were subjected to liquid chromatography (LC)–mass spectrometry (MS) analysis as described previously [25]. Analysis was performed using the Agilent 6130 Series LC/MSD SL system equipped with an Agilent 1260 Infinity Series LC, multi‐ion source, and the ChemStation software (Agilent Technologies, Santa Clara, CA, USA). The sample solution comprised 180 μL of the intercellular lipid extract and 20 μL of an internal standard solution (500 nM N‐heptadecanoyl‐D‐erythro‐sphingosine [Avanti Polar Lipids, Alabaster, AL, USA] in a methanol/2‐propanol/chloroform mixture [9:9:2, v/v/v]). Selected ion monitoring in electrospray ionization mode (as m/z [M‐H]‐) was used to detect ceramides and pCer. A previously described method was used to determine the total protein levels in the SC using half of each tape sample [10]. A BCA kit (Thermo Scientific, Rockford, IL, USA) was used for quantification. Ceramide and pCer levels were normalized to the quantified protein levels.

### 2.4 Skin Capacitance and TEWL Measurements

**FIGURE 2:**
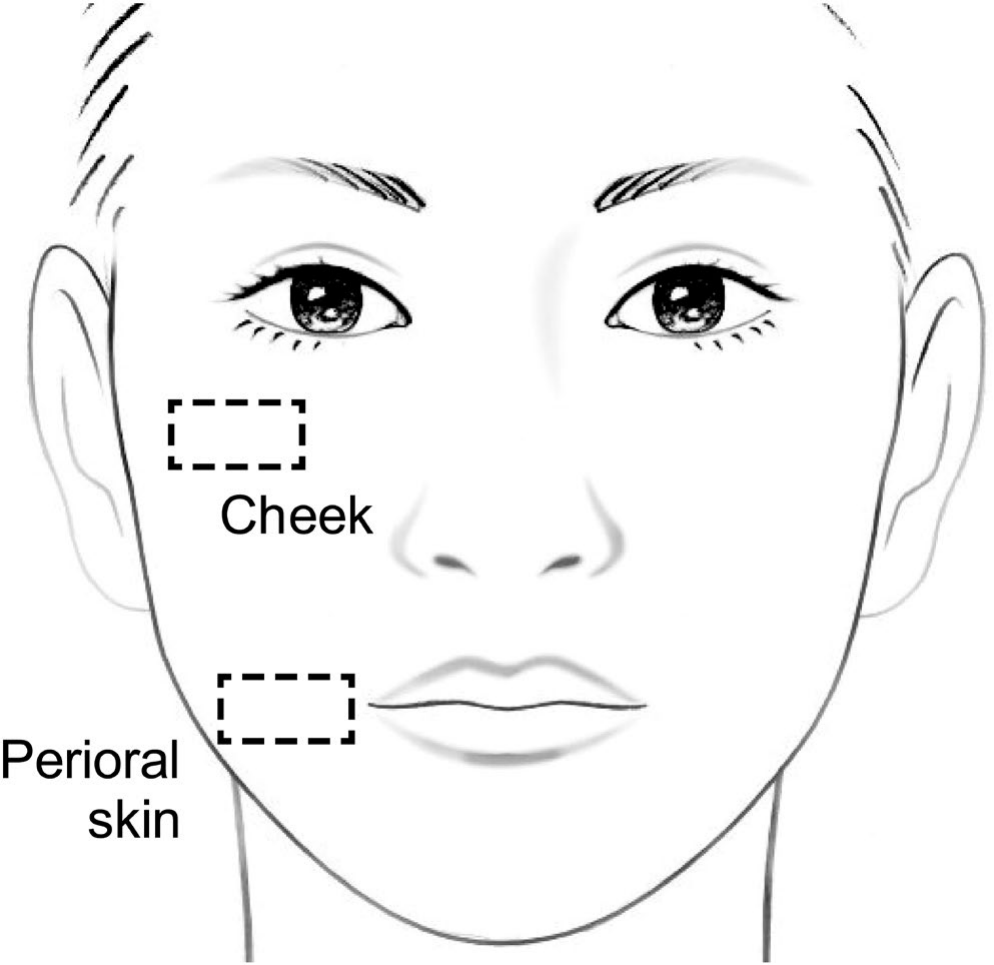
Locations of the cheek and perioral skin.

Corneometer CM825 and Tewameter TM300 (Courage + Khazaka Electronic GmbH, Köln, Germany) were used to measure skin capacitance and TEWL, respectively. These measurements were performed at the cheek and corner of the mouth (perioral skin) (Figure 2).

### 2.5 Corneocyte Surface Area

Using previously described methods [26], human SC specimens were collected from the perioral skins of the participants by stripping with CAF tape (Toppan Co. Ltd., Tokyo, Japan). The tape was tightly pressed onto a glass slide. The glass slide was immersed in xylene overnight to dissolve adhesive glue and remove the tape. The corrected corneocytes were stained with 1% crystal violet and 0.5% brilliant green aq., followed by microscopic observation and imaging. The average projected surface area of the corneocytes at 30 corneocyte locations was calculated and analyzed.

### 2.6 Skin Scaling

One author evaluated the visual scaling scores. KH‐8700 (HYROX Co. Ltd., Tokyo, Japan) was used to obtain magnified images of the skin surface. Measurements were performed at the cheek and perioral skin. The scaling score was determined using a 5‐point scale: 1 = normal, 2 = slight, 3 = moderate, 4 = marked, and 5 = severe, based on the figures shown in a previous study [27].

Score 1: Normal skin: No signs of scaling or flaking.

Score 2: Slight flaking: Slight but definite roughness, with a powdery or ashy appearance.

Score 3: Moderate flaking/scaling: Moderate roughness; somewhat coarse surface.

Score 4: Marked scaling/slight fissuring: Marked roughness, with coarse scaling and evident cracking as uplifted scales.

Score 5: Severe scaling/fissuring: Marked roughness, with very coarse scaling and cracking progressing to fissuring.

### 2.7 Statistical Analysis

GraphPad Prism ver. 9.5.1 (GraphPad Software, MDF Co. Ltd., San Diego, CA, USA) was used to perform all statistical analyses in this study. Skin parameters and the degree of changes in each measurement were analyzed using the test product. If the distribution was considered normal, a paired *t* test or Student's *t* test was performed. In contrast, if the distribution was not normal, Wilcoxon's signed‐rank test was performed. Spearman's correlation coefficient analysis was performed to compare the relationship among physician skin findings, instrumental measurements of skin properties, and questionnaire data. *p* values < 0.05 are considered statistically significant.

## 3 Results

### 3.1 Endogenous Ceramide and pCer Absorption Levels

**FIGURE 3:**
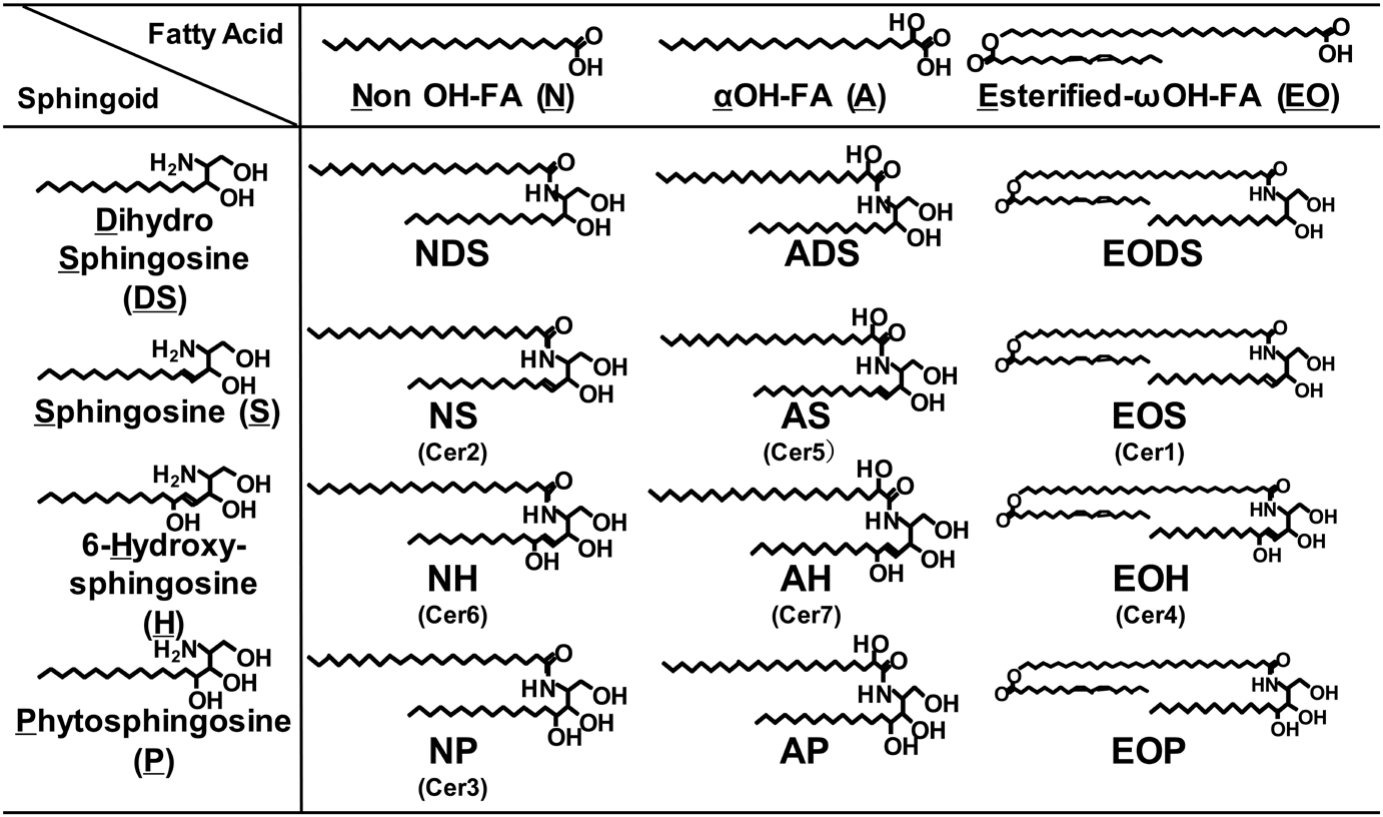
Chemical structures of different ceramide species.

**FIGURE 4:**
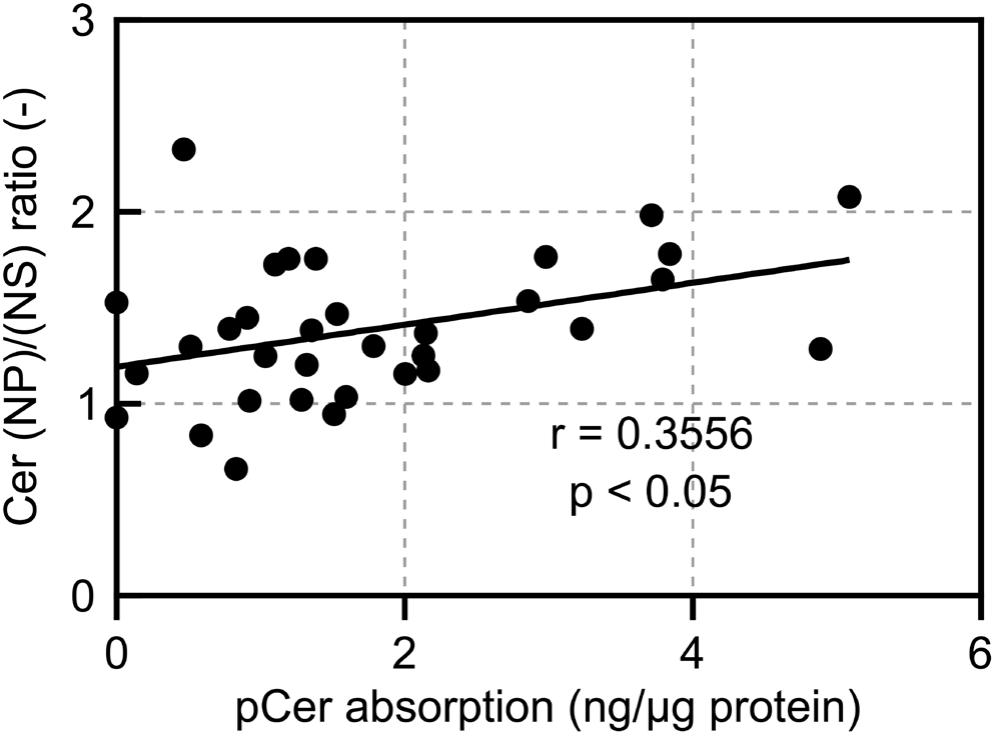
Relationship between pCer absorption level into the SC and Cer (NP)/(NS) ratio after 4 weeks of test spray application (correlation coefficient *r* = 0.3556 *p* < 0.05 via Spearman's correlation coefficient). pCer, pseudo‐ceramide; SC, stratum corneum; Cer, ceramide.

In this study, one participant from each test spray and control spray group dropped out because of adverse events caused by transient sensory irritation. Using LC–MS, the 12 endogenous ceramide species (Figure 3) have been previously described [20], and pCer absorption levels were measured in the collected intercellular lipids of the SC of the participants in the test spray group (Table 4). No significant changes were observed in any of the endogenous ceramide species levels before and after topical spray application twice daily for 4 weeks; however, pCer absorption was observed after 4 weeks of topical application. Evaluation of the correlation between the ceramide (NP)/(NS) ratio, an indicator of SC barrier function [9], and the pCer absorption level revealed a significant positive correlation between the two (Figure 4).

**TABLE 4:** Changes in the different ceramide classes in the SC.

	0 weeks		4 weeks	
Ceramide	Mean	SE	Mean	SE
NDS	0.385	0.015	0.373	0.016
NS	1.107	0.044	1.130	0.050
NH	1.516	0.079	1.551	0.100
NP	1.553	0.092	1.604	0.117
ADS	0.118	0.004	0.120	0.005
AS	0.928	0.042	0.979	0.044
AH	1.499	0.064	1.592	0.078
AP	0.931	0.049	0.970	0.057
EODS	0.137	0.008	0.160	0.014
EOS	0.589	0.041	0.717	0.061
EOH	0.471	0.032	0.540	0.041
EOP	0.104	0.006	0.115	0.009
Total	9.338	0.402	9.851	0.525
PCer	0.000	0.000	1.791	0.233
	(ng/μg protein)			

*Note:* Each ceramide class of the test spray group (*n* = 33) was analyzed via LC–MS 0 and 4 weeks after application. Data are represented as the mean ± SE. One participant dropped out because of adverse events caused by transient sensory irritation.

Abbreviation: SC, stratum corneum.

### 3.2 SC Moisture Retention and Barrier Functions

**FIGURE 5:**
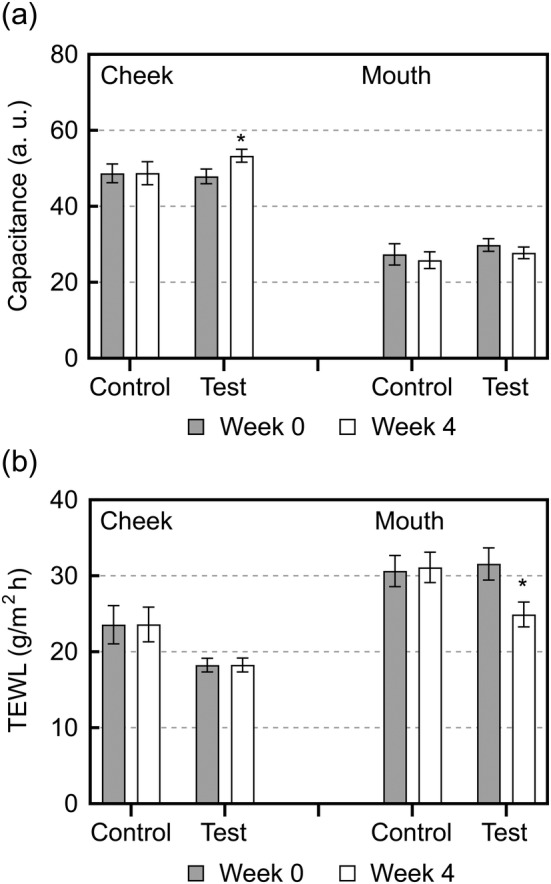
Changes in (a) capacitance and (b) TEWL in the cheek and perioral skin after topical application of the test or control spray. Gray columns represent values before application, and open columns represent values after application for 4 weeks. Data are represented as the mean ± SD. *p < 0.05 versus 0 weeks, via a paired *t* test. TEWL; transepidermal water loss, W0; Week 0, W4; Week 4.

Figure 5a,b demonstrates changes in the SC water content and TEWL in the cheeks and perioral skin before and after 4 weeks of topical application twice daily. Following topical test spray application, the SC water content significantly increased in the cheek. However, no changes in the SC water content were observed in the control spray group before and after topical application.

Topical test spray application significantly decreased TEWL in the perioral skin. However, no changes were observed in the control spray group after topical application.

**FIGURE 6:**
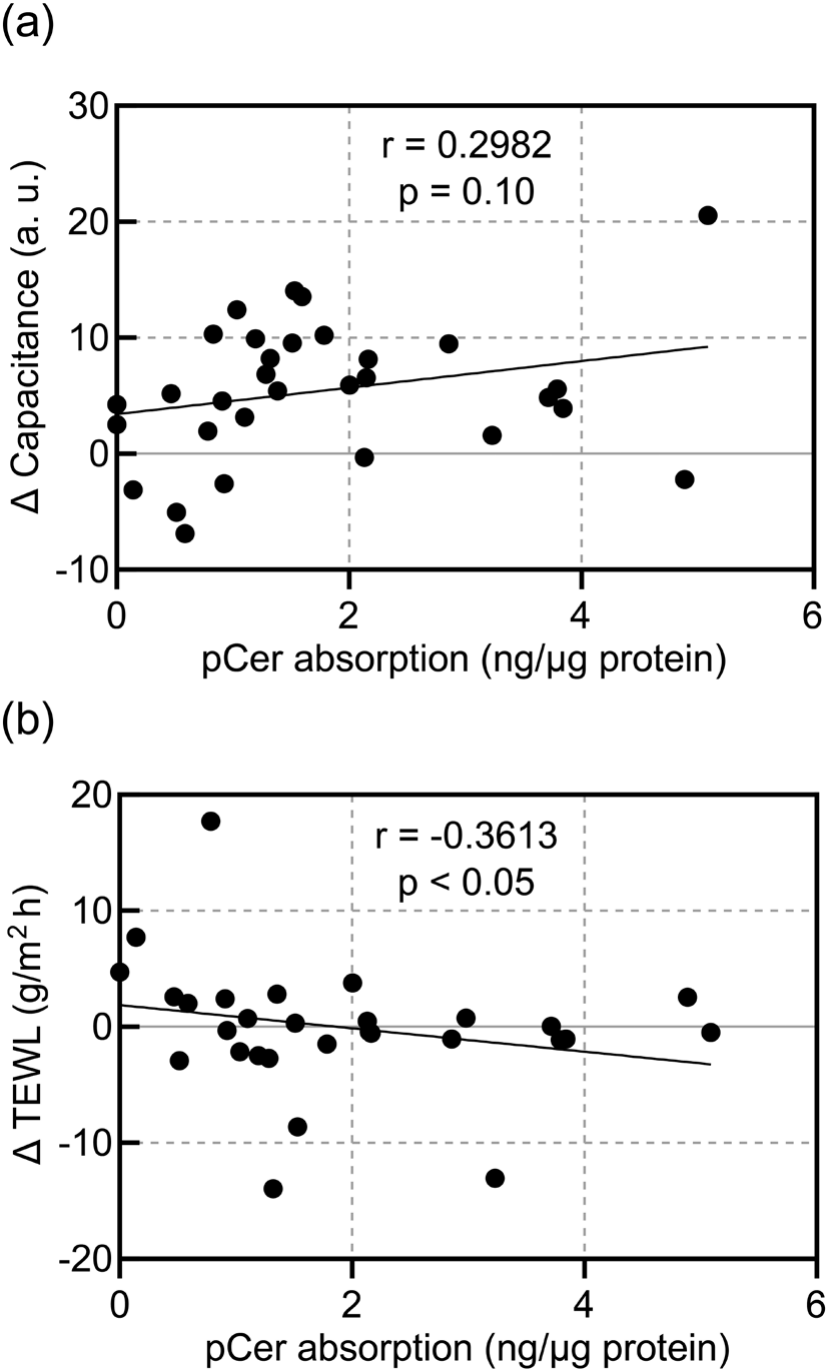
Relationship between pCer absorption level into the SC and each skin condition parameter. (a) Changes in capacitance (correlation coefficient *r* = 0.2982 *p* = 0.10 using Spearman's correlation coefficient). (b) Changes in TEWL (correlation coefficient *r* = −0.3613 *p* < 0.05 using Spearman's correlation coefficient). pCer, pseudo‐ceramide; SC, stratum corneum; and TEWL, transepidermal water loss.

Subsequently, we correlated the pCer absorption level and changes in the SC water content (Δ capacitance) or TEWL (Δ TEWL) after 4 weeks of topical application twice daily (Figure 6a,b). The Δ capacitance increased proportionally to the pCer application level; however, this correlation was not significant. Moreover, ΔTEWL and the pCer absorption level were significantly negatively correlated.

**FIGURE 7:**
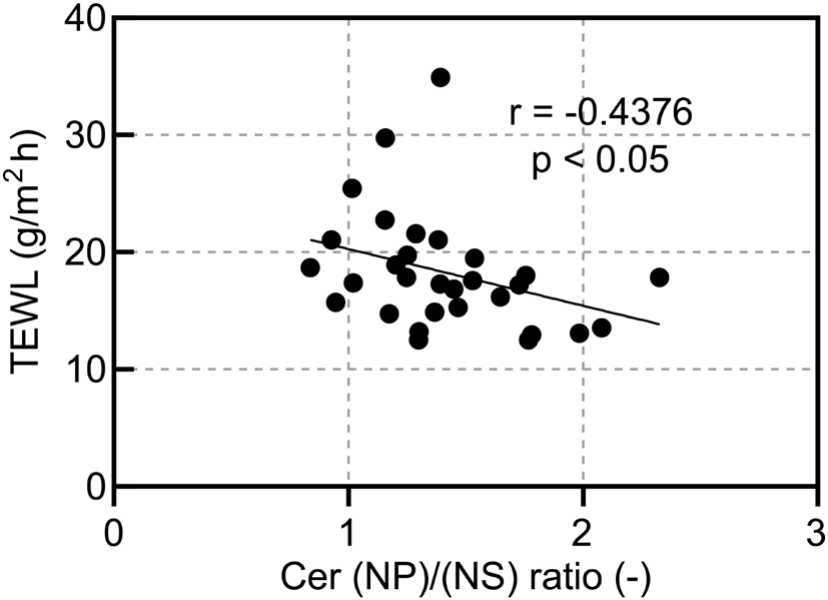
Relationship between the Cer (NP)/(NS) ratio and TEWL in the cheek after 4 weeks of test spray application (correlation coefficient *r* = −0.4376 *p* < 0.05 via Spearman's correlation coefficient). Cer, ceramide; TEWL, transepidermal water loss.

In addition, we correlated TEWL in the cheek and the ceramide (NP)/(NS) ratio after 4 weeks of topical application twice daily (Figure 7) and found a significant negative correlation between the two.

### 3.3 Corneocyte Surface Area

**FIGURE 8:**
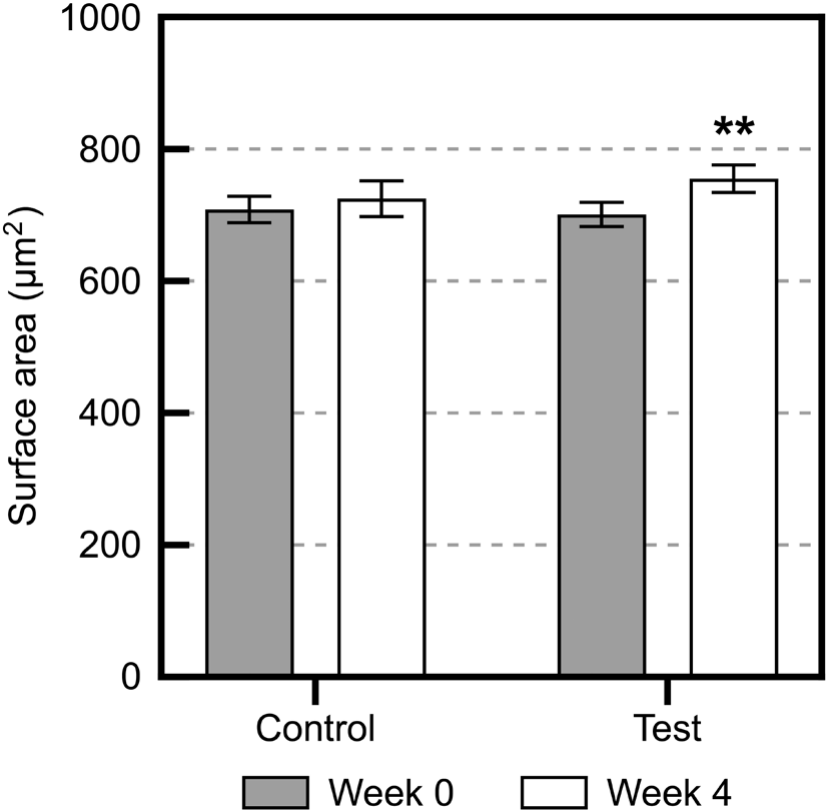
Changes in the surface area of the cheek after topical application of the test or control spray. Gray columns represent values before application, and open columns represent values after application for 4 weeks. **p < 0.01 versus 0 weeks via a paired *t* test.

Figure 8 illustrates the corneocyte surface areas before and after applying the control and test sprays twice daily for 4 weeks. No changes in corneocyte surface area were observed in the control spray group after topical application; however, the surface area significantly increased in the test spray group after topical application.

### 3.4 Scaling Score

No significant visual improvements in the scaling score were observed after topical application of the control spray twice daily for 4 weeks. In contrast, significant visual improvements were observed in the scaling scores of the cheek and perioral skin after topical application of the test spray (Table 5).

**TABLE 5:** Changes in the scaling score after 4 weeks of topical spray application.

Method	Point	Control group (*n* = 19)		Test group (*n* = 33)	
		W0	W4	W0	W4
		Mean ± SE			
Visual evaluation	Cheek	2.92 ± 0.19	2.76 ± 0.24	3.09 ± 0.19	2.38 ± 0.10**
	Mouth	3.03 ± 0.18	2.61 ± 0.16	2.89 ± 0.14	2.36 ± 0.11**

*Note:* Each score was measured before and after treatment for 4 weeks. Control spray group (control group): *n* = 19; test spray group (test group): *n* = 33. Data represent the mean ± SE. **p < 0.01 versus 0 weeks by Wilcoxon's signed rank test; W0, Week 0; W4, Week 4. One participant from each test spray and control spray group dropped out because of adverse events caused by transient sensory irritation.

** *p* < 0.01 (vs. W0).

## 4 Discussion

The efficacies of moisturizer containing pCer that mimics the ceramide (NS) structure [15] targeted the SC barrier and water content functions of atopic dermatitis or sensitive skin with lactic acid irritation were reported in many studies [5, 20, 21, 24]. Ishida et al. [16] proposed that improvements in SC barrier function in atopic dermatitis, whose endogenous ceramide properties are deteriorated, through topical pCer application are associated with an enhancement of the endogenous ceramide profile rather than the ceramide level induced by pCer absorption. A recent study demonstrated that the ceramide level in individuals with skin highly sensitive to lactic acid irritation was not significantly different from that in individuals without sensitive skin; however, the ceramide profile was worse in individuals with sensitive skin.^10^ Therefore, it was expected that pCer absorption is effective for individuals with sensitive skin and impaired barrier function.

In this study, the intercellular lipid content quantified on tape‐stripped SC in the test group that applied a pCer‐containing spray for 4 weeks revealed the presence of pCer in the SC. Takagi et al. demonstrated that this spray formulation exhibits high pCer absorption ability [25]. Furthermore, Ishida et al. [16] demonstrated pCer penetration in the SC of patients with atopic dermatitis after continuous use of a pCer‐containing formulation for 4 weeks. Therefore, pCer could have absorbed into the SC of the participants who applied the pCer spray formulation for 4 weeks in this study, similar to the findings of previous studies.

Previous research has shown that the spray formulation used in this study improves skin dryness and increases ceramide levels of patients with atopic dermatitis [24]. Although endogenous ceramide levels of atopic dermatitis are significantly decreased compared with healthy skin [4], there are no significant differences between those of skin highly sensitive to lactic acid irritation and non‐sensitive skin [10]. So we investigated changes to ceramide profiles in patients without atopic dermatitis. In this study, significant improvements in the SC water content at the cheek and TEWL perioral skin were observed in the test group without elevation in the endogenous ceramide level; however, these changes were not observed in the control group. Moreover, significant improvements were observed in the visually assessed scaling score at the cheek and perioral skin of the test group; however, no changes were observed in the control group. On the basis of these results, pCer‐containing spray is proposed to be valuable for improving the SC function in individuals who have self‐perceived sensitive skin with an impaired epidermal barrier whose endogenous ceramide levels are not significantly decreased compared with healthy controls.

Ceramides that are present in the SC form a barrier by building a multilamellar structure of intercellular lipids; therefore, endogenous ceramide levels and barrier function are strongly correlated [6, 7]. PCer is a synthetic ceramide with optimal structure (comparable to the structure of ceramide NS) and with a moisturizing efficacy equivalent to that of natural ceramides [5, 15]. In addition, Ishida et al. proposed that pCer penetrates and accumulates in the SC of patients with atopic dermatitis, thereby playing an essential role in improving the SC barrier [16]. In the present study, although there was no significant change in the endogenous ceramide levels of participants after topical application of the pCer‐containing spray, a significant negative correlation was observed between the level of pCer absorption in the SC and changes in TEWL. These findings imply that pCer absorbed into the SC contributes to barrier formation in the SC as a ceramide functional ingredient. However, we were unable to observe the behavior of pCer applied to the SC in this study, warranting further investigation. Even though the test spray contained Eucalyptus extract, which stimulates ceramide synthesis in cultured human keratinocytes [21], the ceramide levels of subject in test spray group did not increase. This might be because the subjects did not have low ceramide levels, unlike patients with atopic dermatitis. In the present study, no significant changes were observed in TEWL in the cheek after 4 weeks of topical application; however, significant improvements were observed in TEWL in the perioral skin, whose barrier was impaired more than that of the cheek before treatment. Several studies have revealed that topical application of pCer‐containing formulations improves the barrier function of patients with atopic dermatitis as well as those with sensitive skin [5, 15, 20, 21, 24]. Therefore, pCer absorption into the SC may improve the barrier function of the SC of individuals with self‐perceived sensitive skin and impaired epidermal barrier; however, significant changes were only observed where the barrier function was particularly impaired.

Although there was no significant increase in SC ceramide subclass levels due to topical application of the pCer‐containing spray, the pCer absorption level and ceramide (NP)/(NS) ratio were significantly positively correlated after topical application of the spray. Yokose et al. reported that the ceramide (NP)/(NS) ratio is a representative indicator of the SC ceramide profile because it exhibits a negative correlation with TEWL [9]. A previous study reported that the ceramide (NP)/(NS) ratio significantly decreased in individuals with skin highly sensitive to lactic acid irritation compared to that in individuals without sensitive skin; however, their endogenous ceramide levels did not differ significantly [10]. In this study, the continuous use of the pCer‐containing spray for participants with self‐perceived sensitive skin and impaired epidermal barrier improved SC barrier function (Figure 5b). In addition, changes in TEWL were significantly negatively correlated with pCer absorption (Figure 6b). Moreover, Figure 7 indicates that TEWL and the ceramide (NP)/(NS) ratio in the cheek after 4 weeks of application of the pCer‐containing spray were significantly negatively correlated. This correlation suggests that the ceramide profile of test group after the topical application of pCer‐containing spray is associated with their barrier function, as previously reported [9]. These relationships support the hypothesis that pCer absorption into the SC can qualitatively improve impaired endogenous ceramide profiles, rather than ceramide levels, by ameliorating SC barrier function. These observations suggest that pCer absorption into the SC could improve the skin of atopic dermatitis with ceramide deficiency, as has been previously described [16], as well as sensitive skin with impaired barrier function whose ceramide level is not decreased but whose ceramide profile is not a healthy skin phenotype.

Certain reports indicate that ceramide production in the SC is associated with the keratinization process [8, 9, 28]. The corneocyte surface area, which is an indicator of the SC turnover rate, is significantly smaller in sensitive skin than in non‐sensitive skin due to abnormal keratinization [10, 26]. In the present study, we confirmed that the corneocyte cell area significantly increased upon topical application of the pCer‐containing spray, but no changes were observed in the control group. Therefore, the turnover of the SC of individuals with self‐perceived sensitive skin and impaired epidermal barrier was very likely to be normalized by continuous application of the pCer‐containing spray. Moreover, the correlation of the ceramide profile after 4 weeks of application of the pCer‐containing spray and pCer absorption in this study may be attributed to normalized keratinization by continuous spray application. However, further investigation is needed to verify the relationship between the improvement in barrier function and normalization of keratinization induced by topical application of pCer‐containing moisturizers.

This study has several limitations. First, the subjects were selected based on their skin barrier function. This selection process may have allowed for the inclusion of participants who potentially had atopic dermatitis. Second, the control spray used in this study was a commercially available product, and there were ingredients present in the test spray in addition to pCer that were not included in the control spray. These ingredients may have influenced the results, making it difficult to fully isolate the effects of pCer. Future studies should aim to use a control spray with more standardized ingredients to better clarify the specific impact of pCer in skin treatments.

In conclusion, we have demonstrated the efficacy of a pCer‐containing spray in improving SC function in individuals with impaired epidermal barrier function and self‐perceived sensitive skin. An intriguing aspect of this observation is that these improvements were achieved not through the restoration of endogenous ceramide levels, but rather through topical application of pCer similar to endogenous ceramides. Additionally, the findings suggest that pCer absorption into the SC is valuable for skin with a deteriorated endogenous ceramide profile, including sensitive skin with impaired barrier function. Therefore, our study provides new insights into the pathophysiology of barrier function in sensitive skin.

## Author Contributions

D.W. designed the study and acquired the data. T.A., D.W., K.T., and E.S. analyzed the data. T.A. wrote the original manuscript. K.K. revised the manuscript. All authors contributed to the study and approved the submitted version of the manuscript.

## Ethics Statement

This study was conducted in compliance with the relevant ethical policies and was approved by the Ethical Review Committee of Kao Corporation on February 1, 2019 (approval number: D053‐181214).

## Consent

All participants provided written informed consent during the enrollment process.

## Conflicts of Interest

The authors declare no conflicts of interest.

## Data Availability

Data sharing is not applicable to this article as no new data were created or analyzed in this study.
